# Effect of theory of mind and peer victimization on the schizotypy–aggression relationship

**DOI:** 10.1038/npjschz.2016.1

**Published:** 2016-03-23

**Authors:** Bess Y H Lam, Adrian Raine, Tatia M C Lee

**Affiliations:** 1 Laboratory of Neuropsychology, The University of Hong Kong, Hong Kong, China; 2 Departments of Criminology, Psychiatry, and Psychology, University of Pennsylvania, Philadelphia, PA, USA; 3 Laboratory of Cognitive Affective Neuroscience, The University of Hong Kong, Hong Kong, China; 4 The State Key Laboratory of Brain and Cognitive Sciences, The University of Hong Kong, Hong Kong, China; 5 Institute of Clinical Neuropsychology, The University of Hong Kong, Hong Kong, China

## Abstract

Prior longitudinal studies have established the relationship between schizophrenia and violence. However, previous studies on aggression and schizotypal personality are scarce. The present study examines whether peer victimization mediates the relationship between schizotypy and reactive-proactive aggression, and whether theory of mind (ToM) moderates this mediation. Schizotypy, peer victimization, reactive-proactive aggression, and ToM were assessed in 237 undergraduates. Peer victimization mediated the relationship between schizotypy and reactive aggression. ToM moderated this mediation effect; although peer victimization partially explains the schizotypy–aggression relationship, higher ToM skills weakened the detrimental effect of schizotypy on peer victimization which in turn reduces reactive aggression. In contrast, the moderated mediation was not significant for the proactive aggression model. Findings help delineate the underlying mechanism of the relationship between schizotypy and aggression. It is suggested that aggression could be reduced by enhancing ToM skills, thereby reducing peer victimization and the resultant schizotypy.

## Introduction

Prior longitudinal studies have established a relationship between schizophrenia and aggression.^1^ To better understand the mechanisms underlying this relationship, recent work^2,3^ has shed more light on non-clinical individuals with schizotypal personality traits who are at risk for schizophrenia-spectrum disorders.^4–6^ Schizotypy is a multi-dimensional construct including interpersonal skill deficits, unusual cognitive-perceptual experiences as well as disorganized features.^7^ Nevertheless, the factors underlying the link between schizotypy and aggression are not widely studied. Investigating such a relationship and the underpinnings at the non-clinical level might provide further implications for the prevention of, and intervention for, aggression and schizotypal symptomatology.

Recent studies have found that schizotypy is associated with aggression in adults^3,8^ and youth.^9^ For example, Seah and Ang^3^ suggested that schizotypy is associated with two forms of aggression, namely reactive and proactive aggression. Reactive aggression, which is associated with hostile attribution bias,^10^ unusual perceptual experiences and ideas of reference,^8^ is defined as a response to provocation or a perceived threat.^11^ On the other hand, proactive aggression, which is associated with positive outcome expectancy,^10^ psychopathic personality and blunted affect,^8^ is defined as goal-oriented and calculated aggression performed to obtain external reward.^12^ These two forms of aggression are closely related to each other, yet they are distinguishable and can co-occur in the same individual.^13^ For instance, prior study has already validated and confirmed this two-factor aggression construct.^14^

However, previous studies^9,15^ investigating the underlying factors of the schizotypy–aggression relationship are scarce. Moreover, subjects have varied from children to adults across these studies. Specifically, Fanning *et al*.^15^ found that perceived threat mediated the relationship between psychosis proneness (perceptual aberrations, magical ideation and social anhedonia) and aggressive behaviors in undergraduates. Since this study considered three aspects of aggression (aggressive history, aggressive tendencies and laboratory aggression) as one single dependent variable without specifying the form of aggression, this raises a question about the generalizability of findings to more specific forms of aggression.

In this context, Raine *et al.*^9^ investigated the relationship between schizotypy and reactive-proactive aggression in children aged 8–16 years. Schizotypy was almost three times more strongly related to reactive aggression compared with proactive aggression. After controlling for reactive aggression, proactive aggression was no longer related to schizotypy. Furthermore, peer victimization mediated the schizotypy–reactive aggression relationship, accounting for 58.3% of the common variance between these constructs. Findings suggest that any aggression–schizotypy relationship is specific to reactive forms of aggression, and that victimization is one mechanism explaining this relationship. Although this was the first study to identify any mediator of the schizotypy–aggression relationship, it remains to be seen whether these findings generalize to young adults, and whether this mediation holds at all levels of social-cognitive functioning, such as theory of mind.

Social-cognitive functioning which includes theory of mind, emotion recognition, and social perception is related to aggression.^16^ Also, this functioning is impaired in schizophrenia.^17,18^ For instance, Zhu *et al.*^18^ found that people with schizophrenia scored worse in the eye gaze discrimination task and the faux pas recognition task in comparison with the healthy controls. Similar impairment was also found in early psychosis who were within 2 years of their first psychotic episode.^19^ In terms of the sub-clinical population, a recent study found similar results.^20^ Specifically, they found that the people who were at clinical high risk for psychosis had worse theory of mind and social perception when compared with those of healthy controls. Therefore, it can be hypothesized that it may have a significant role in the schizotypy–peer victimization–aggression relationship. The present study focused on one social-cognitive function, theory of mind (ToM). Specifically, ToM refers to the ability to infer the mental status of others, and to understand and predict of behavior based on these representations.^21^ Moreover, it is crucial to decode and understand social cues and hence essential to clarify the development of adaptive social behavior.^22,23^

Previously, Renouf *et al.*^16^ found that reactive and proactive aggression was associated with ToM as well as peer victimization in children. Reactive aggression was associated with low ToM and proactive aggression was associated with high ToM, especially in those who experienced high levels of peer victimization. One longitudinal study found that poor ToM predicted that children would become a victim, bully, or bully-victim in early adolescence.^24^ Taken together, these findings suggested that deficits in ToM, which facilitate healthy social relationships, pose a risk for peer victimization as well as aggression in children and adolescents.

Although the prior literature has found impaired ToM in patients with schizophrenia,^17,18^ questions remain. How is it related to schizotypy? Is the association between schizotypy, peer victimization and aggression also applicable to young adults with differing ToM skills? In terms of the relationship between schizotypy and ToM, inconsistent results have been found in adults. While Langdon and Coltheart^25^ found that high-schizotypy groups performed worse at sequencing false-belief stories, Fernyhough *et al*.^26^ did not find an association between schizotypy and ToM. Inconsistencies across studies may be attributable to different measurement tools for ToM and schizotypy. In addition, the psychometric properties of ToM measures are understudied.^27^ Similarly, findings regarding ToM and peer victimization are also inconsistent. For example, ToM was found to be unrelated to peer victimization in children aged 4–6 years,^28^ while Shakoor *et al.*^24^ found the opposite results in children and adolescents.

Taken altogether, the relationship between schizotypy, ToM, peer victimization, and aggression is yet to be clearly delineated. In this context, the present study aims to test these associations by adopting measurement tools for schizotypy and ToM with reliable psychometric properties in healthy individuals.^29,30^ Furthermore, we aim to examine whether the relationship between schizotypy, peer victimization, and aggression varies across individuals with different levels of ToM skills with cross-sectional data. It is hypothesized that:

1. Schizotypy will be associated with reactive, but not proactive aggression after controlling for all covariates and reactive/proactive aggression;
2. Peer victimization will mediate the relationship between schizotypy and reactive–proactive aggression in young adults such that schizotypy will pose a risk for peer victimization, which in turn will increase the risk for reactive aggression;
3. Theory of mind will moderate the schizotypy-peer victimization-aggression mediation such that the higher the level of ToM, the weaker the association between schizotypy and peer victimization.

## Results

### Preliminary group comparisons and correlations

Female participants were less reactively aggressive and victimized by their peers compared with males (*P<*0.05). Age was positively related to reactive aggression and peer victimization (*P<*0.05) (Table 1). Consequently, age, sex, employment status and education were analyzed as covariates in the following mediation and moderation analyses.

### Tests of mediation

**Figure 1:**
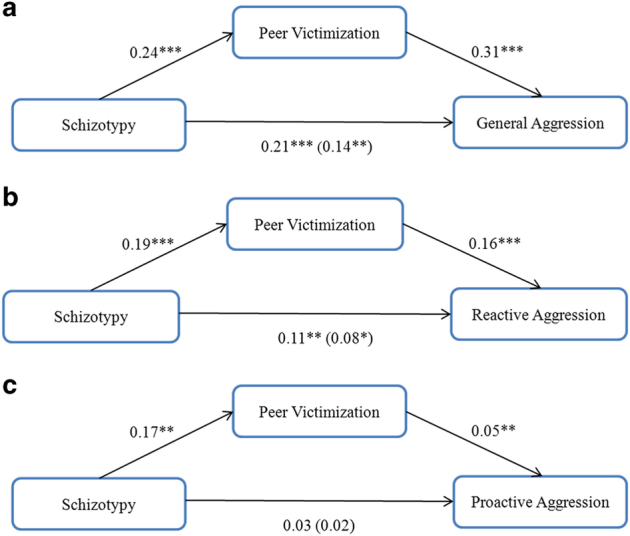
The mediation between schizotypy, peer victimization and (**a**) general aggression (mean indirect effect=0.071, 95% CI=0.0326, 0.1291), (**b**) reactive aggression (mean indirect effect=0.03, 95% CI=0.0096, 0.0637), and (**c**) proactive aggression (mean indirect effect=0.01, 95% CI=0.0005, 0.0238) after controlling for age, sex, education and employment status (coefficient in parenthesis in the figure indicated the direct effect after mediated by peer victimization). *P⩽0.05, **P⩽0.01, ***P⩽0.001. CI, confidece interval.

Figure 1 presents the results for hypothesis 1 and 2 (simple mediation). Schizotypy was positively associated with peer victimization (*P<*0.05) when general aggression, reactive, or proactive aggression were the dependent variables in the mediation. Peer victimization was positively associated with general, reactive, and proactive aggression, controlling for schizotypy and all covariates (*P*⩽0.01). The 95% confidence intervals (CIs) showed that the effect of schizotypy on general, proactive or reactive aggression was partially mediated by peer victimization except that schizotypy was not significantly associated with proactive aggression after controlling for reactive aggression and other covariates (*P*>0.05; Figure 1). Specifically, peer victimization mediated the schizotypy–aggression and the schizotypy–reactive aggression relationship, accounting for 33.3 and 27.6% of the common variance between these constructs, respectively.

### Tests of moderated mediation

#### Theory of mind and general aggression

**Figure 2:**
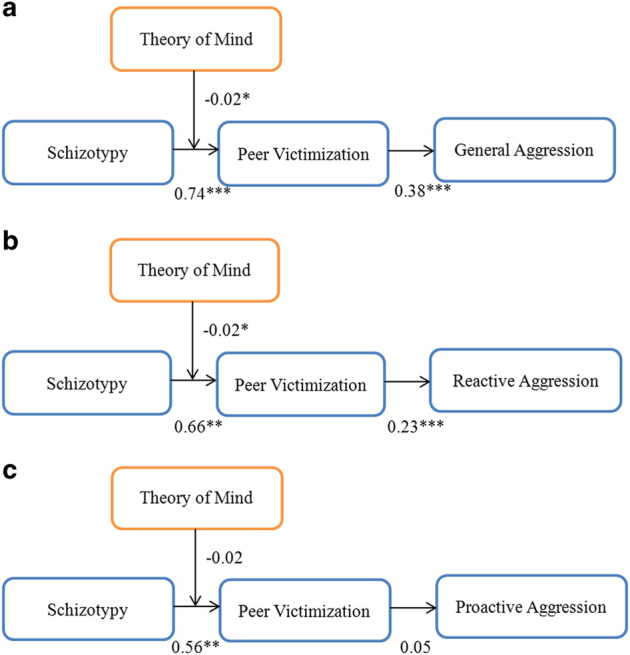
The moderated mediation of schizotypy, peer victimization, theory of mind, and (**a**) general aggression (*P<*0.05). *Post hoc* analysis results at the mean faux pas total score (mean indirect effect=0.10, 95% CI=0.0518, 0.1743), and one s.d. above (mean indirect effect=0.06, 95% CI=0.0115, 0.1253) and below the mean (mean indirect effect=0.15, 95% CI=0.0597, 0.2723) were significant; (**b**) reactive aggression (*P<*0.05). *Post hoc* analysis results at the mean faux pas total score (mean indirect effect=0.05, 95% CI=0.0206, 0.0959), and one s.d. above (mean indirect effect=0.03, 95% CI=0.0022, 0.0684) and below the mean (mean indirect effect=0.08, 95% CI=0.0253, 0.1540) were significant; and (**c**) proactive aggression (*P*>0.05) after controlling for age, sex, education, and employment status. Significance tests for the indirect effects were based on biascorrected confidence intervals derived from 5,000 bootstrapped samples (Shrout and Bolger^36^). *P⩽0.05, **P⩽0.01, ***P⩽0.001.

As predicted in hypothesis 3, the positive association between schizotypy and peer victimization was weaker in those with higher total faux pas scores (FP) when compared with those with lower scores. Figure 2a reports the overall and *post hoc* analysis results for the mediation moderated by FP. The 95% CIs for the *post hoc* analysis as well as the interaction of schizotypy with faux pas total score (SPQ×FP) in predicting peer victimization demonstrated that the conditional indirect effect was significantly moderated by the values of FP (*P<*0.05). Overall, the indirect effects of schizotypy through peer victimization on general aggression decreased from 0.15 to 0.06 with the increasing values of FP (*P<*0.05; Figure 2a).

### Tests of moderated mediation

#### Theory of mind and reactive–proactive aggression

To further test how ToM moderated the relationship between schizotypy, peer victimization, and reactive/proactive aggression, the two forms of aggression were studied using the proposed moderated mediation model (Figure 2b,c). Figure 2b, c reports the overall and *post hoc* analysis results for the mediation model moderated by ToM in predicting the two subtypes of aggression, respectively. The 95% CI for the *post hoc* analysis as well as the interaction of schizotypy with faux pas total score (SPQ×FP) in predicting peer victimization was significant for reactive aggression model (*P<*0.05). Overall, the indirect effects of schizotypy through peer victimization on reactive aggression decreased from 0.08 to 0.03 with the increasing values of FP (Figure 2b). In contrast, the moderated mediation was not significant for the proactive aggression model (*P*>0.05; Figure 2c).

### Alternative model

Given this was a cross-sectional study using self-report measures in which we cannot claim causality, we also tested an alternative model with peer victimization as the independent variable, schizotypy as the mediator, theory of mind as the moderator and general aggression as the dependent variable (Supplementary Figure 1). This alternative moderated mediation model was not significant (*P*>0.05), suggesting that the proposed moderated mediation model of the present study (Figure 2) is a more parsimonious explanation of the schizotypy–aggression relationship and its mediation.

## Discussion

The current study aimed to examine whether schizotypy was more closely related to reactive than proactive aggression and whether peer victimization mediated the relationship between schizotypy and reactive–proactive aggression in young adults. Moreover, it aimed to investigate whether ToM moderated such a mediation. Overall findings supported the *a priori* hypotheses, showing that schizotypy was more related to reactive aggression and that the mediation model of schizotypy–peer victimization–aggression also applied to young adults.^9^ Importantly, ToM was found to moderate this mediation effect, in line with prior literature.^16^ These findings suggested that peer victimization partially explains the schizotypy–aggression relationship, and that higher ToM skills mitigate the effect of schizotypy on peer victimization, which in turn reduced one’s aggression, particularly reactive aggression. This suggests, in theory, the possibility of reducing reactive aggression in individuals with schizotypal personality traits by targeting their theory of mind skills.

Consistent with hypothesis 1, peer victimization mediated the relationship between schizotypy and reactive (but not proactive) aggression in young adults, a finding in line with prior work.^9^ After controlling for the covariates, those with higher schizotypy scores were more vulnerable to being victimized by their peers, and this victimization experience in turn was associated with reactive aggression. This mediation also withstood controlling for proactive aggression, documenting a robust and selective relationship for reactive aggression. As predicted, schizotypy was not associated with proactive aggression through peer victimization after controlling for reactive aggression and the covariates. The dissociative findings for reactive and proactive aggression suggest that the underlying mechanisms for these two subtypes of aggression are different in young adults.^9^

Theory of mind significantly moderated the schizotypy—peer victimization—aggression mediation. The mediation between schizotypy, peer victimization and aggression was significantly weakened for those with higher ToM ability compared with those with lower ToM ability. This was consistent with our *a priori* hypothesis and prior findings by Renouf *et al.*^16^ Indeed, ToM is essential in positive social interactions,^22,23,31,32^ and as such it is not surprising that ToM has a significant role in mitigating the effect of schizotypy and peer victimization on aggression.

In order to efficiently intervene or prevent aggression committed by people with schizotypy, it may be helpful to identify how ToM skills have a significant role in predisposing to reactive aggression. Findings of the present study revealed that ToM moderated the mediation model with reactive aggression but not proactive aggression as the dependent variable. These suggest that training a schizotypal individual’s ToM, particularly the ability in recognizing the occurrence of a faux pas as well as in understanding the mental states of the others during social interactions, could potentially reduce the effect of schizotypy in predisposing to peer victimization and reactive aggression. Overall, the moderation of ToM was only significant for the reactive aggression model, a finding consistent with the *a priori* hypothesis that schizotypal individuals are more likely to aggress in a more reactive, impulsive manner than in a proactive planned manner.^9^

Although the findings of the present study are consistent with prior literature, it is noteworthy that schizotypy is not the only personality construct related to the propensity for reactive–proactive aggression. For instance, antisocial personality disorder is found to be positively related to both reactive and proactive aggression while borderline personality disorder (alone or comorbidly with antisocial personality disorder) is associated with more reactive aggression.^33^ Future studies may compare the aggression propensity and ToM between different types of personality constructs or disorders in order to delineate the distinct association of aggression and ToM with schizotypy and other related personality constructs, respectively.

It might be argued that the expression of personality traits including schizotypal personality traits vary across culture. Therefore, it is essential to employ a measurement tool for personality that is well validated across cultures so that the findings can be generalised to various cultural groups. In light of this concern, the present study employed SPQ-B which is a well-validated measure for schizotypy in different cultures (Western and Asian).^34,35^ Nevertheless, future studies should be cautious in assessing personality traits and drawing conclusions from related findings.

The current study has a number of limitations. First, this was a cross-sectional study using self-report measures, and as such there are limits on making causal inferences. Mediation models can help support a causal model, but they cannot establish causality. However, findings lay a conceptual foundation for future longitudinal studies. Moreover, an alternative statistical model with the bootstrapping method^36^ was analyzed to address this limitation as well as the single-informant biases. Indeed, this bias-corrected bootstrap which was employed in the present study is suggested to enhance the power of the mediation analyses.^37^ Nevertheless, there are minor drawbacks of the bootstrapping method that need to be cautious about. For instance, it may include slight inconsistency while replicating the same experiment with the same data given that it is based on random resampling variability. Second, the current sample consisted of Chinese young adults from Hong Kong and consequently findings cannot be generalized to other racial or ethnic groups. Third, findings are specific to individual differences in schizotypy and cannot as yet be generalized to clinical schizotypy. Future studies could usefully address these three limitations. Last but not least, we did not employ a specific measurement tool (e.g., IQ test) for participants’ verbal ability which may affect one’s ToM performance in the present study because of the time constraints. Although their education level and age that are indicators of verbal ability were controlled for in the analyses, it is suggested to measure participants’ verbal ability using measures such as IQ test in future ToM studies.

In spite of these limitations, the present study has several strengths. In contrast to clinical studies of schizophrenia, we examined individual differences in schizotypal individuals in the normal population in order to avoid the possible confounding effects of factors associated with psychiatric illness (e.g., medication). More importantly, findings help to confirm that the mediation relationship between schizotypy, peer victimization, and aggression also applies to young adults, and that ToM moderates such a relationship.

There are several implications of these findings. First, the significant moderated mediation effect of peer victimization and ToM on the schizotypy–aggression relationship implies that both cognitive and social processes are important in explaining the aggression committed by schizotypal individuals.^2^ In addition, the significant role played by peer victimization and impaired ToM in such a relationship suggests that schizophrenia–spectrum disorders could be prevented by minimizing patients’ peer victimization experiences and increasing their ability to infer others’ mental states. Taken together, peer victimization reduction programs^38^ as well as social cognition training programs such as Social Cognition and Interaction Training (SCIT)^39^ might help to prevent or intervene in aggression in schizotypy. Although the peer victimization reduction programs may be mostly targeting younger children and adolescents, they can potentially be tailor made for schizotypy.

## Materials and methods

### Participants

Two hundred and forty seven bilingual undergraduate students were recruited in Hong Kong in the present study. All participants were recruited through mass email advertisement and Introduction to Psychology Course at The University of Hong Kong. The final analysis included 237 participants (males: 31.2%; females: 68.8%) who met the following inclusion criteria in order to be included in the present study: (1) had never been diagnosed with schizophrenia or any other psychiatric disorders by Diagnostic and Statistical Manual of Mental Disorders (DSM- IV-TR);^40^ (2) were not on any current psychiatric medications; and (3) were 18 years old or above. The large proportion of female participants (68.8%) in the present study might be explained by the male:female ratio (3:7) of the social sciences undergraduate students at The University of Hong Kong. The mean age was 18.92 years (s.d.=1.16 years), ranging from 18 to 25 years. The majority of the participants were first year undergraduate students (*n*=183, 77.2%; Supplementary Table 1). University Institutional Review Board (IRB) approval was obtained at the Human Research Ethics Committee for Non-clinical Faculties of The University of Hong Kong. All participants were informed of the general nature of this study before their written informed consent was obtained. Participants were asked about their demographic information (e.g., age) and assessed with the measures below. Upon completion, participants were rewarded with class credit for the Introduction to Psychology course and were debriefed about the purpose of the study.

### Measures

All of the following self-report measures were translated and back-translated from English to Chinese.

### Peer victimization

The Multidimensional Peer Victimization Scale^41^ was used to assess different forms of peer victimization: physical victimization, social manipulation, verbal victimization, and attack on property. Participants were asked to rate each item on a Likert scale, ranging from 0 (not at all) to 2 (more than once). Total score (the summation of all items) was used for the final analysis (*α*=0.74).

### Reactive–proactive aggression

The self-report Reactive-Proactive Questionnaire (RPQ)^2^ was used to assess reactive and proactive aggression. Participants rated their own aggressive behaviors on a Likert scale from 0 (never) to 2 (often). The RPQ consists of two subscales: reactive aggression and proactive aggression. Construct validity, criterion validity, convergent validity and discriminant validity have been established.^2^ The internal reliabilities were 0.74 (total), 0.69 (reactive), and 0.59 (proactive) in the present study.

### Schizotypy

Schizotypal personality traits were measured by the Schizotypal Personality Questionnaire-Brief (SQP-B).^42^ The SPQ-B (present study *α*=0.70) is a short form of the full 74-item SPQ^43^ measuring cognitive perceptual, interpersonal, and disorganized features (see Supplementary Table 2 for the intercorrelations of SPQ-B features).^7^

### Theory of mind

The Faux Pas Test (FP)^44^ which consists of 10 faux pas and 10 control stories was used to measure ToM. Since FP has been used to measure ToM in healthy adult participants in the Chinese population with good reliabilities,^45^ it was adopted in the present study. Faux pas refers to a speaker saying something that he or she should not have said, not knowing or realizing that these words are not appropriate in the context. There were a total of six questions (FP Q1—Q6) for each story. Specifically, FP Q1 measured the participants’ ability in detecting a faux pas (detection); FP Q2 assessed their ability in identifying the person who committed the faux pas (identification); FP Q3 assessed their ability in interpreting the mental state of the recipient; FP Q4 assessed their ability in understanding the speaker’s intention; FP Q5 and Q6 were control questions measuring their ability in recalling specific story content. For each correct answer for the 10 faux pas stories, 1 point would be given and 0 points otherwise. Initially, four sub-scores (FP Q1- Q4) were computed.^45^ However, with the present data, the correlations of FP Q4 with FP Q1–3 ranged from 0.20 to 0.21, while in contrast the inter-correlations among FP Q1–3 ranged from 0.88 to 0.97. Hence, FP Q4 was not included in final analyses and the total score of ToM (FP) was computed by the summation of FP Q1–3. Thus, the total score of ToM (FP) ranged from 0 to 30 points. Higher scores referred to higher ability of ToM. Previously, the total score of FP (summation of FPQ1-Q3) was 17.64 (s.d.=6.15) in the healthy adults (mean age=41.3 years) in prior study.^45^ The Chinese version of the test has a 3-month test–retest reliability of.83 and an interrater reliability of.76.^18^ The overall reliability of FPwas.88 for the present study.

### Statistical analyses

Pearson’s correlations, one-way analysis of variance and *t*-tests were initially performed for the demographic information and major variables in the present study. To examine the relationship between schizotypy, ToM, peer victimization and aggression, mediation, and moderated mediation analyses were performed using the SPSS PROCESS macro^46^ and employing the bootstrapping method as outlined by Shrout and Bolger.^36^ The bootstrapping method was applied in order to avoid power problems due to asymmetric and other non-normal sampling distributions of an indirect effect.^47^ Schizotypy was the independent variable; ToM was the moderator; peer victimization was the mediator; and reactive-proactive aggression were the dependent variables. A confidence interval that does not contain zero indicates significant mediation (*P*<0.05).^36^ The rule-of-thumb for adequate sample size (*N*⩾50+8×variables) suggests that at least 74 participants would be needed for the analysis of the present study. Our present sample size met this threshold. Non-centered predictors and interaction terms were used in the following analyses.

## Figures and Tables

**Table 1:** Intercorrelations between study variables

	*1*	*2*	*3*	*4*	*5*	*6*
Schizotypy	—					
Peer victimization	0.29***	—				
General aggression	0.30***	0.42***	—			
Reactive aggression	0.28***	0.38***	0.95***	—		
Proactive aggression	0.23***	0.33***	0.68***	0.43***	—	
Faux pas total score (FP)	0.03	−0.04	−0.04	−0.06	0.02	—
Total	7.89	3.41	5.11	4.62	0.49	22.26
s.d.	4.30	3.51	3.05	2.47	1.02	5.73
Mode	10	0	6	5	0	30
Range	0–26	0–17	0–21	0–14	0–8	6–30
Kurtosis	0.57	0.83	3.33	0.95	18.01	−0.69

***P⩽0.001.

## References

[bib1] Fazel, S. , Gulati, G. , Linsell, L. , Geddes, J. R. & Grann, M. Schizophrenia and violence: systematic review and meta-analysis. PLoS Med. 6, 1–15 (2009). 10.1371/journal.pmed.1000120 PMC2718581 19668362

[bib2] Raine, A. Schizotypal personality: neurodevelopmental and psychosocial trajectories. Annu. Rev. Clin. Psychol. 2, 291–326 (2006). 17716072 10.1146/annurev.clinpsy.2.022305.095318

[bib3] Seah, S. L. & Ang, R. P. Differential correlates of reactive and proactive aggression in Asian adolescents: relations to narcissism, anxiety, schizotypal traits, and peer relations. Aggressive Behav. 34, 553–562 (2008). 10.1002/ab.20269 18506675

[bib4] Claridge, G. Single indicator of risk for schizophrenia: probable fact or likely myth? Schizophr. Bull. 20, 151–168 (1994). 8197412 10.1093/schbul/20.1.151

[bib5] Miller, P. , Byrne, M. , Hodges, A. , Lawrie, S. M. & Johnstone, E. Childhood behaviour, psychotic symptoms and psychosis onset in young people at High Risk of Schizophrenia; Early findings from the Edinburgh High Risk Study. Psychol. Med. 32, 173–179 (2002). 11883726 10.1017/s0033291701004779

[bib6] Siever, L. J. et al. Cognitive and brain function in schizotypal personality disorder. Schizophr. Res. 54, 157–167 (2002). 11853990 10.1016/s0920-9964(01)00363-2

[bib7] Raine, A. et al. Cognitive-perceptual, interpersonal, and disorganized features of schizotypal personality. Schizophr. Bull. 20, 191–201 (1994). 8197415 10.1093/schbul/20.1.191

[bib8] Raine, A. et al. The Reactive–Proactive Aggression Questionnaire: differential correlates of reactive and proactive aggression in adolescent boys. Aggressive Behav. 32, 159–171 (2006). 10.1002/ab.20115 PMC2927832 20798781

[bib9] Raine, A. , Fung, A. L. C. & Lam, B. Y. H. Peer victimization partially mediates the schizotypy—aggression relationship in children and adolescents. Schizophr. Bull. 37, 937–945 (2011). 21795613 10.1093/schbul/sbr082 PMC3160209

[bib10] Schwartz, D. et al. Social- cognitive and behavioral correlates of aggression and victimization in boys’ play groups. J. Abnorm. Child. Psychol. 26, 431–440 (1998). 9915650 10.1023/a:1022695601088

[bib11] Dodge, K. A. , Lochman, J. E. , Harnish, J. D. , Bates, J. E. & Pettit, G. S. Reactive and proactive aggression in schoolchildren and psychiatrically impaired chronically assaultive youth. J. Abnorm. Psychol. 106, 37–51 (1997). 9103716 10.1037//0021-843x.106.1.37

[bib12] Fite, P. J. , Colder, C. R. , Lochman, J. E. & Wells, K. C. Developmental trajectories of proactive and reactive aggression from fifth to ninth grade. J. Clin. Child Adolesc. Psychol. 32, 412–421 (2008). 10.1080/15374410801955920 18470777

[bib13] Dodge K. A . in The Development and Treatment of Childhood Aggression (eds Pepper D. J. & Rubin K. H.) pp. 201–218 (Erlbaum, Hillsdale, NJ, 1991).

[bib14] Fung, A. L. C. , Raine, A. & Gao, Y. Cross-cultural generalizability of the reactive- proactive aggression questionnaire (RPQ). J. Pers. Assess 91, 473–479 (2009). 19672753 10.1080/00223890903088420

[bib15] Fanning, J. R. , Berman, M. C. , Mohn, R. S. & McCloskey, M. S. Perceived threat mediates the relationship between psychosis proneness and aggressive behavior. Psychiatry Res. 186, 210–218 (2011). 20965573 10.1016/j.psychres.2010.09.010 PMC3041859

[bib16] Renouf, A. et al. Interactive links between theory of mind, peer victimization, and reactive and proactive aggression. J. Abnorm. Child Psychol. 38, 1109–1123 (2010). 20544385 10.1007/s10802-010-9432-z PMC3283569

[bib17] Frith, C. D. The positive and negative symptoms of schizophrenia reflect impairments in the perception and initiation of action. Psychol Med. 17, 631–648 (1987). 3628624 10.1017/s0033291700025873

[bib18] Zhu, C. Y. et al. Impairments of social cues recognition and social functioning in Chinese people with schizophrenia. Psychiat. Clin. Neurol. 61, 149–158 (2007). 10.1111/j.1440-1819.2007.01630.x 17362432

[bib19] Langdon, R. , Connors, M. H. , Still, M. , Ward, P. B. & Catts, S. Theory of mind and neurocognition in early psychosis: a quasi-experimental study. BMC Psychiatry 14, 316 (2014). 25472859 10.1186/s12888-014-0316-6 PMC4263012

[bib20] Barbato, M. et al. Theory of mind, emotion recognition and social perception in individuals at clinical high risk for psychosis: findings from the NAPLS-2 cohort. Schizophr. Res. Cognit. 2, 133–139 (2015). 10.1016/j.scog.2015.04.004 PMC5041592 27695675

[bib21] Premack, D. & Woodruff, G. Does the chimpanzee have a ‘theory of mind’? Behav. Brain Sci. 4, 515–526 (1978).

[bib22] Astington, J. W. The future of theory-of-mind research: Understanding motivational states, the role of language, and real-world consequences. Commentary on ‘Meta-analysis of theory-of-mind development: the truth about false belief’. Child Dev. 72, 685–687 (2001). 11405572 10.1111/1467-8624.00305

[bib23] Hughes, C. & Leekam, S. What are the links between theory of mind and social relations? Review, reflection, and new directions for studies of typical and atypical development. Soc. Dev. 13, 590–619 (2004).

[bib24] Shakoor, S. et al. A prospective longitudinal study of children’s theory of mind and adolescent involvement in bullying. J. Child Psychol. Psychiatry 53, 254–261 (2012). 22081896 10.1111/j.1469-7610.2011.02488.x PMC3272094

[bib25] Langdon, R. & Coltheart, M. Mentalizing, schizotypy and schizophrenia. Cognition 71, 43–71 (1999). 10394709 10.1016/s0010-0277(99)00018-9

[bib26] Fernyhough, C. , Jones, S. R. , Whittle, C. , Waterhouse, J. & Bentall, R. P. Theory of mind, schizotypy, and persecutory ideation in young adults. Cogn. Neuropsychiatry 13, 233–249 (2008). 18484289 10.1080/13546800801936516

[bib27] Harrington, L. , Langdon, R. , Siegert, R. & McClure, J. Schizophrenia, theory of mind and persecutory delusions. Cogn. Neuropsychiatry 10, 87–104 (2005). 16571454 10.1080/13546800344000327

[bib28] Badenes, L. V. , Estevan, R. A. C. & Bacete, F. J. G. Theory of mind and peer rejection at school. Soc. Dev. 9, 271–283 (2000).

[bib29] Lee, T. M. C. et al. Faux pas deficits in people with medial frontal lesions as related to impaired understanding of a speaker's mental state. Neuropsychologia 48, 1670–1676 (2010). 20156464 10.1016/j.neuropsychologia.2010.02.012

[bib30] Mata, I. , Mataix-Cols, D. & Peralta, V. Schizotypal Personality Questionnaire-Brief: factor structure and influence of sex and age in a non-clinical population. Pers. Indiv. Differ. 38, 1183–1192 (2005).

[bib31] Dodge, K. A. Social cognition and children’s aggressive behavior. Child Dev. 51, 162–170 (1980). 7363732

[bib32] Mitchell, P . Introduction to Theory of Mind. Children, Autism and Apes. Arnold, (1997).

[bib33] Ross, J. M. & Babcock, J. C. Proactive and reactive violence among intimate partner violent men diagnosed with antisocial and borderline personality disorder. J. Fam. Violence 24, 607–617 (2009).

[bib34] Raine, A. , Fung, A. L. C. & Lam, B. Y. H. Peer victimization partially mediates the schizotypy-aggression relationship in children and adolescents. Schizophr. Bull. 37, 937–945 (2011). 21795613 10.1093/schbul/sbr082 PMC3160209

[bib35] Reynolds, C. A. , Raine, A. , Mellingen, K. , Venables, P. H. & Mednick, S. A. Three-factor model of schizotypal personality: invariance across culture, gender, religious affiliation, family adversity, and psychopathology. Schizophr. Bull. 26, 603 (2000). 10993401 10.1093/oxfordjournals.schbul.a033481

[bib36] Shrout, P. E. & Bolger, N. Mediation in experimental and nonexperimental studies: new procedures and recommendations. Psychol. Methods 7, 422 (2002). 12530702

[bib37] Hayes A. F. , Scharkow M . The relative trustworthiness of inferential tests of the indirect effect in statistical mediation analysis: does method really matter? Psychol. Sci. 24: 1918–1927. (2013). 23955356 10.1177/0956797613480187

[bib38] Leadbeater, B. & Hoglund, W. Changing the social contexts of peer victimization. J Can Acad Child Adolesc Psychiatry 15, 21–46 (2006). 18392192 PMC2277274

[bib39] Penn, D. L. , Roberts, D. L. , Combs, D. & Sterne, A. The development of the Social Cognition and Intervention Training program for schizophrenia spectrum disorders. Psychiatr. Serv. 58, 449–451 (2007). 17412842 10.1176/ps.2007.58.4.449

[bib40] American Psychiatric Association. Diagnostic and Statistical Manual of Mental Disorders, 4th edn (Author, Washington, DC, 2000).

[bib41] Mynard, H. & Joseph, S. Development of the multidimensional peer-victimization scale. Aggressive Behav. 26, 169–178 (2000).

[bib42] Raine, A. & Benishay, D. The SPQ-B: a brief screening instrument for schizotypal personality disorder. J. Pers. Disord. 9, 346–355 (1995).

[bib43] Raine, A. The SPQ: a scale for the assessment of schizotypal personality based on DSM-III-R criteria. Schizophr. Bull. 17, 555–564 (1991). 1805349 10.1093/schbul/17.4.555

[bib44] Stone, V. E. , Baron-Cohen, S. & Knight, R. T. Frontal lobe contributions to theory of mind. J. Cogn. Neurosci. 10, 640–656 (1998). 9802997 10.1162/089892998562942

[bib45] Lam, B. Y. H. , Raine, A. & Lee, T. M. C. The relationship between neurocognition and symptomatology in people with schizophrenia: social cognition as the mediator. BMC Psychiatry 14, 138 (2014). 24885177 10.1186/1471-244X-14-138 PMC4026589

[bib46] Hayes, A. F . Introduction to Mediation, Moderation, and Conditional Process Analysis: A Regression-based Approach (Guilford Press, 2013).

[bib47] MacKinnon, D. P. , Lockwood, C. M. & Williams, J. Confidence limits for the indirect effect: distribution of the product and resampling methods. Multivar. Behav. Res. 39, 99–128 (2004). 10.1207/s15327906mbr3901_4 PMC2821115 20157642

