# Supplementary material for: Effect of theory of mind and peer victimization on the schizotypy–aggression relationship
Source: NPJ Schizophr. 2016 Mar 23;2:16001–. doi: 10.1038/npjschz.2016.1 (PMC4898892; doi:10.1038/npjschz.2016.1)
Supplement: Supplementary Tables [file npjschz20161-s1.pdf]

Supplementary material

Table 1

Undergraduate academic level of participants.

Undergraduate academic level	N	%
Year 1	183	77.2
Year 2	34	14.3
Year 3	18	7.6
Year 4	2	0.8
Total	237	100

Table 2

Inter-correlations between study variables.

	1	2	3
1 Schizotypy subscale- interpersonal	-		
2 Schizotypy subscale- cognitive perceptual	0.27***	-	
3 Schizotypy subscale- disorganized	0.45***	0.26***	-
3 Peer victimization	0.19**	0.19**	0.27***
4 Reactive aggression	0.20**	0.17*	0.28***
5 Proactive aggression	0.15*	0.15*	0.22***
6 Faux pas total score (FP)	-0.01	0.08	-0.03
Internal reliability	.70	.28	.68

*p <= 0.05.
**p <= 0.01.
***p <=0.001.
