# Supplementary figures and images for: Effect of theory of mind and peer victimization on the schizotypy–aggression relationship
Source: NPJ Schizophr. 2016 Mar 23;2:16001–. doi: 10.1038/npjschz.2016.1 (PMC4898892; doi:10.1038/npjschz.2016.1)

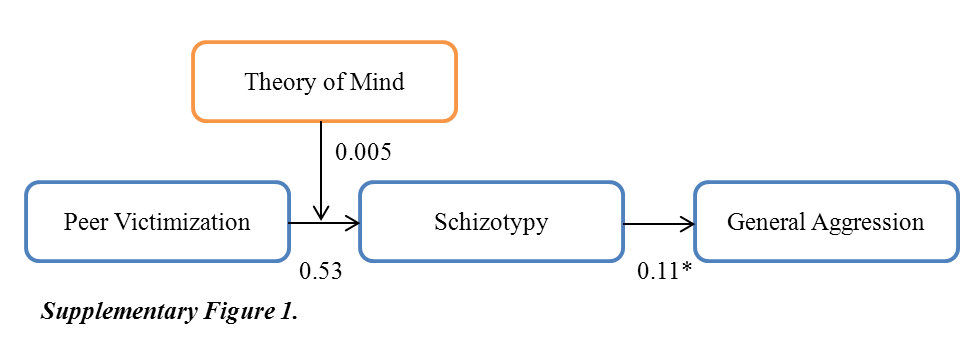

Supplement: Supplementary Figure 1 [file npjschz20161-s2.tiff]
